# Cerebrospinal fluid, antineuronal autoantibody, EEG, and MRI findings from 992 patients with schizophreniform and affective psychosis

**DOI:** 10.1038/s41398-020-00967-3

**Published:** 2020-08-12

**Authors:** Dominique Endres, Sophie Meixensberger, Rick Dersch, Bernd Feige, Oliver Stich, Nils Venhoff, Miriam Matysik, Simon J. Maier, Maike Michel, Kimon Runge, Kathrin Nickel, Horst Urbach, Katharina Domschke, Harald Prüss, Ludger Tebartz van Elst

**Affiliations:** 1 Section for Experimental Neuropsychiatry, Department of Psychiatry and Psychotherapy, Medical Center - University of Freiburg, Faculty of Medicine, University of Freiburg, 79104 Freiburg, Germany; 2 Department of Psychiatry and Psychotherapy, Medical Center - University of Freiburg, Faculty of Medicine, University of Freiburg, 79104 Freiburg, Germany; 3 Clinic of Neurology and Neurophysiology, Medical Center - University of Freiburg, Faculty of Medicine, University of Freiburg, 79106 Freiburg, Germany; 4 Medical Care Center, Neurology, 78464 Konstanz, Germany; 5 Department of Rheumatology and Clinical Immunology, Medical Center - University of Freiburg, Faculty of Medicine, University of Freiburg, 79106 Freiburg, Germany; 6 Department of Neuroradiology, Medical Center - University of Freiburg, Faculty of Medicine, University of Freiburg, 79106 Freiburg, Germany; 7 Center for Basics in Neuromodulation, Faculty of Medicine, University of Freiburg, 79106 Freiburg, Germany; 8 Department of Neurology and Experimental Neurology, Charité - Universitätsmedizin Berlin, 10117 Berlin, Germany; 9 German Center for Neurodegenerative Diseases (DZNE) Berlin, 10117 Berlin, Germany

**Keywords:** Schizophrenia, Diagnostic markers

## Abstract

The central role played by cerebrospinal-fluid (CSF) examinations including antineuronal autoantibody (Ab) testing is increasingly recognized in psychiatry. The rationale of this study was to present a multimodally investigated group of patients. In total, 992 patients were analyzed for CSF alterations: 456 patients with schizophreniform and 536 with affective syndromes. Ab measurement included testing for established antineuronal IgG-Abs against intracellular antigens in serum (Yo/Hu/Ri/cv2[CRMP5]/Ma1/Ma2/SOX1/TR[DNER]/Zic4/amphiphysin/GAD65) and for cell surface antigens in the CSF (NMDAR/AMPA-1/2-R/GABA-B-R/LGI1/CASPR2/DPPX). In 30 patients with “red flags” for autoimmune psychosis, “tissue tests” were performed. Additional diagnostics included MRI and EEG analyses. CSF white-blood-cell counts were increased in 4% and IgG indices in 2%; CSF-specific oligoclonal bands were detected in 4%; overall, 8% displayed signs of neuroinflammation. In addition, 18% revealed increased albumin quotients. Antineuronal Abs against intracellular antigens were detected in serum in 0.6%. Antineuronal Abs against established cell surface antigens were detected in serum of 1% and in the CSF of 0.3% (CSF samples were only questionably positive). Abnormal IgG binding in “tissue tests” was detected in serum of 23% and in CSF of 27%. In total, 92% of the Ab-positive patients demonstrated at least one sign of brain involvement in additional diagnostics using CSF, MRI, EEG, and FDG-PET. In summary, CSF basic analyses revealed signs of blood–brain-barrier dysfunction and neuroinflammation in relevant subgroups of patients. Established antineuronal IgG-Abs were rare in serum and even rarer in the CSF. “Tissue tests” revealed frequent occurrences of Ab-binding; therefore, novel antineuronal Abs could play a relevant role in psychiatry.

## Introduction

In the last decade, the study of autoimmune encephalitis (AE) and autoimmune psychosis (AP) has rapidly developed^1,2^, largely due to the discovery of anti-N-methyl-D-aspartate receptor (NMDAR) encephalitis in 2007. Prior to this discovery, patients with suspected AE were mainly tested for antineuronal autoantibodies (Abs) against intracellular antigens in the context of paraneoplastic processes^3^. Since 2007, however, the importance of AE has increased in the field of psychiatry with the recognition that anti-NMDAR encephalitis often manifests with psychotic symptoms and that these patients are usually seen initially by psychiatrists^4–7^. At the same time, several other antineuronal Abs against cell surface antigens (e.g., LGI1) have been discovered that are associated with psychiatric symptoms. These Abs seem to play a direct pathophysiological role and can occur non-paraneoplastically^8^. The discovery of new Abs is expected in the future; therefore, in this respect, screening examinations using unfixed rodent tissue sections can be helpful for Ab detection^9,10^. Nevertheless, at present, large investigations of the prevalence of antineuronal Abs in patients with psychoses have been limited to unimodal studies using serum^11–13^. Smaller investigations of cerebrospinal fluid (CSF) have revealed antineuronal Abs at significantly lower numbers when compared to serum analyses^14,15^. Multimodal studies that include electroencephalography (EEG), magnetic resonance imaging (MRI), and especially CSF basic analyses with quantification of antineuronal Abs are lacking at present. CSF diagnostics play a central role in the context of antineuronal Abs, since low antigen-specific immunoglobulin (Ig) G Ab titers can be detected even rarely in the serum of healthy individuals^11–13^. This finding emphasizes the necessity of evaluating the pathophysiological relevance of these Abs by CSF analyses^2,7,16^. In addition, extended diagnostics using EEG, MRI, or [^18^F] fluorodeoxyglucose positron emission tomography (FDG-PET) would allow the detection or exclusion of brain involvement in sero-positive patients. According to recently published international consensus criteria for AP, a diagnosis of “probable AP” requires typical CSF, MRI, or EEG findings, while confirmation of a diagnosis of “definite AP” requires the detection of IgG antineuronal Abs in the CSF^2^.

### Rationale

At the Department of Psychiatry and Psychotherapy of the University Hospital in Freiburg, patients with schizophreniform syndromes have routinely been offered lumbar punctures (LPs) since approximately June 2009, based on new developments and personal experiences with AEs/APs^14,17,18^. The aim of the present study was to conduct a retrospective evaluation of a large cohort of patients with schizophreniform and affective syndromes who underwent multimodal examinations consisting of CSF analyses, antineuronal Ab testing in serum/CSF, EEG, and MRI.

## Patients and methods

The study received approval from the local ethics committee of the University of Freiburg (EK Fr 396/18). All patients gave written informed consent before LP. Between January 2006 and November 2019, 992 patients were included in the present study.

### Patient cohort

All inpatients with schizophreniform syndromes (according to the International Statistical Classification of Diseases and Related Health Problems criteria, version 10 [ICD-10]: F20.X–F29.X, F06.0-2, F10.5-F19.5) and affective syndromes (unipolar depression following ICD-10: F32.X, F33.X, F06.3 and bipolar disorder following ICD-10: F30.X, F31X, F06.3) who underwent an LP at the Department of Psychiatry and Psychotherapy were included. Patients who were transferred to the Department of Neurology for further investigations were not included. Only the first LP results were analyzed for each patient. The patients were clinically diagnosed by experienced senior psychiatrists according to the ICD-10 criteria. For statistical analyses, patients were classified according to their predominant psychiatric syndromes. Patients with schizophreniform or affective syndromes who were also diagnosed with dementia were excluded (ICD-10: F00.X-F04.X). Other preexisting (e.g., earlier stroke) or newly described (e.g., migraine) neurological comorbidities were recorded but not considered as an exclusion criteria if the LP was performed within the diagnostic process of the psychiatric disorder. Since approximately June 2009, patients with schizophreniform syndromes have been offered CSF analysis routinely at our institution, whereas patients with affective syndromes have not been examined routinely. Clinical data were extracted from the patient discharge letters. Some parameters were also taken from the basic clinical documentation, such as the Clinical Global Impression (CGI; ref. ^19^), Global Assessment of Functioning (GAF; ref. ^20^), and psychopathological scores following the German Association for Methodology and Documentation in Psychiatry (“AMDP-scores”; ref. ^21^).

### Laboratory methods

#### CSF analyses

CSF and serum samples were collected simultaneously from all patients^22^. All CSF/serum samples were analyzed in the CSF laboratory of the local department of neurology (https://www.uniklinik-freiburg.de/neurologie/klinik/diagnostische-einrichtungen/liquor-labor.html). The basic CSF analysis included the determination of white blood cell (WBC) counts (ref.: <5/µL), total protein (ref.: <450 mg/L), age-related albumin quotients (AQs; ref.: <40 years: <6.5 × 10^−3^; 40–60 years: <8 × 10^−3^; >60 years: <9.3 × 10^−3^), IgG indices (ref.: <0.7), and oligoclonal bands (OCBs) in serum and/or CSF. The OCBs were evaluated as positive if present at ≥2 in CSF with none in the serum (“Wurster type II”) or if present at more than 2 in the CSF than in the serum (“Wurster type III”). A correction for WBC counts was made if the WBC count was increased due to blood contamination (correction formula: 1 cell/µL of WBC count reduction per 1000 red blood cells/µL). The detailed methodology has been described in previous papers from the working group^14,23–26^.

#### Antineuronal antibodies against intracellular and thyroid antigens

An immunoassay of serum samples has been performed since 2006 at our institution for the detection of antineuronal IgG Abs against intracellular antigens (https://www.ravo.de/de/Produkte/Line_Assays.php). Initially, Abs against nine antigens were analyzed (Yo, Hu, Ri, Cv2/CRMP5, Ma1, Ma2, SOX1, amphiphysin, and GAD65; ravo PNS+2 Blot®, Freiburg, Germany). Since mid-2014, Abs against TR(DNER) and Zic4 were added (ravo PNS 11 Line Assay®, Freiburg, Germany). Weak bands are questionably positive and rated (+), while clearly positive bands are rated (+++). CSF tests were only performed in selected cases (e.g., in unclear cases with positive serum results). Anti-thyroid Abs against thyroid peroxidase (TPO), thyroglobulin (TG), and thyroid-stimulating hormone receptor (TSHR) were analyzed using electrochemiluminescence immunoassay tests (Roche, Basel, Switzerland).

#### Antineuronal antibodies against cell surface antigens

The analysis with fixed biochip assays has been established since 2011 (Euroimmun-kits®, Lübeck, Germany). This initially involved the testing of IgG Abs against five antigens (NMDAR, AMPA-1/2-R, GABA-B-R, LGI1, CASPR2). In 2018, testing for Abs against DPPX was added (“mosaic 6” from Euroimmun®; Lübeck, Germany). The tests were initially performed exclusively in the CSF, but since approximately January 2016, both CSF and serum samples have been routinely analyzed. Prior to that date, combined CSF and serum samples were only conducted in particular cases. The Ab findings were divided into questionably positive (+), slightly positive (++), and clearly positive (+++). From 2006 to 2011 (and later in special cases), material was sent to the reference laboratory at John Radcliffe Hospital (Prof. Vincent, Oxford, United Kingdom) for anti-NMDAR IgG Abs testing using live cell assays and for anti-VGKC IgG Abs testing using RIAs. The results of these tests are already published^14,24^. For reasons of consistency, the results of the previously published work are listed in the results section; additional unsystematic investigations at Oxford for individual cases are not analyzed here. Testing for Abs associated with demyelinating diseases (AQP4 and MOG) has been established since 2018 on our ward for schizophreniform psychosis using Euroimmun® biochip kits (Lübeck, Germany). Since the end of 2018, tissue-based assays using indirect immunofluorescence on unfixed murine brain tissue were established in patients with “red flags” for AP (e.g., catatonia or CSF specific OCBs)^27,28^ (Prof. Prüss, Charité and DZNE, Berlin, Germany; see exemplary in^9^). Only positive (+++) IgG antibody binding patterns were included in the analysis.

### Instrument-based diagnostics

#### EEG

All patients were offered an EEG examination on admission. The EEGs included a resting-state EEG for approximately ten minutes and (if possible) a hyperventilation (HV) phase for ~3 min. The EEGs were evaluated by the responsible physicians. In addition, an automated detection of intermittent generalized rhythmic delta/theta activity (IRDAs/IRTAs) was performed. The methodology has been described in the previous papers^29,30^, and the findings were divided into pre-HV-, post HV-, HV-difference (post-HV–pre-HV), and overall-IRDAs/IRTAS.

#### MRI

The MRI protocol included at least T1-weighted (axial 5 mm thick fast spin echo slices on a 1.5 Tesla, MPRAGE sequence with isotropic 1 mm^3^ voxels on a 3 Tesla scanner), DWI (axial 5 mm thick slices), and FLAIR sequences (coronal 3 mm thick fast spin echo slices on a 1.5 Tesla, 3D SPACE sequence with isotropic 1 mm^3^ voxels on a 3 Tesla scanner). The evaluation was performed by experienced senior physicians in neuroradiology.

### Available datasets

Due to the retrospective approach, not all parameters were available for all patients; moreover, the procedures have been continuously optimized and adapted over the past years. The available datasets are presented in Table 1.

**Table 1:** Overview of the examined parameters and number of patients examined.

	Parameters	Total *N* (Schizophreniform/affective syndrome)
*Testing in Serum and CSF*		
Anti-thyroid antibodies	Antigens: TSHR, TPO, TG	Serum: 530 (274/256)
Established antineuronal IgG antibodies against different cell surface antigens	Antigens: NMDA-R, AMPA-1/2-R, GABAB-R, LGI1, CASPR2, DPPX^a^	Serum: 475 (216/259), CSF: 741 (359/382)
Testing for IgG anti-NMDAR and anti VGKC- complex antibodies (Prof. Vincent, Oxford, UK)	Antigens: NMDA-R, VGKC	Serum: 39 (29/10)^b^
“Tissue tests” (Prof. Prüss, Berlin, Germany)	Antineuronal Ab testing using indirect immune-fluorescence on unfixed murine brain tissue	Serum and CSF: 30 (16/14)
Established antineuronal IgG antibodies against different intracellular antigens	Antigens: Yo, Hu, CV2/CRMP5, Ri, Ma1/2, SOX1, GAD65, amphiphysin, Tr^c^, Zic4^c^	Serum: 826 (405/421)
Established antineuronal IgG antibodies associated with demyelinating diseases	Antigens: MOG, AQP4	Serum: 102 (67/35)
Basic CSF analyses	White blood cell count, total protein, albumin quotient, IgG index, OCBs in serum/CSF	CSF overall: 992 (456/536); [WBC: 982 (454/528), protein: 991 (455/536), AQ: 989 (456/533), IgG Index: 989 (456/533), OCBs in serum: 965 (449/516), OCB in CSF: 966 (449/517)]
*Instrument-based diagnostics*		
EEG	Resting state, hyperventilation period ➢ Classification: Normal, continuous generalized slow activity, continuous regional slow activity, intermittent generalized slow activity, intermittent regional slow activity, epileptic pattern	954 (449/505), 803 (396/407)
MRI of the brain	T1/MPRAGE/DWI/FLAIR ➢ Classification: Normal, non-specific white matter changes (punctuate or patchy and/or confluent), gray matter changes (with special consideration of amygdalae, hippocampi, and other limbic structures), (post-) inflammatory lesions, atrophic changes (global or local), macroangiopathic vascular alterations, microhemorrhages, cysts, tumors, anatomical variants, and other changes	896 (418/478)

*CSF* cerebrospinal fluid, *WBC* white blood cell, *AQ* albumin quotient, *OCB* oligoclonal bands, *IgG* immunoglobulin G, *EEG* electroencephalography, *MRI* magnetic resonance imaging. *Ab* antibody, *AE* autoimmune encephalitis, *AMDP* association for methodology and documentation in psychiatry, *AMPA* α-amino-3-hydroxy-5-methyl-4-isoxazolepropionic acid, *ANCOVA* analysis of covariance, *AP* autoimmune psychosis, *AQP4* aquaporin-4, BBB blood–brain-barrier, *CASPR2* Contactin-associated protein-like 2, *CGI* clinical global impression, *CRMP5* collapsin response mediator protein 5, *DNER* Delta/Notch-like epidermal growth factor-related receptor, *DPPX* dipeptidyl-peptidase-like protein-6, *FDG-PET* [^18^F] fluorodeoxyglucose positron emissiontomography, *FLAIR* fluid attenuated inversion recovery, *GABA* γ-aminobutyric acid, *GAD65* Glutamat-decarboxlase 65 kD, *GAF* global assessment of functioning, *Hu* Initials of first patient diagnosed, *HV* Hyperventilation, *ICD* International Classification of Diseases, *Ig* immunglobulin, *IgLON5* Iglon family member 5, *IRDAs/IRTA* Intermittent generalized rhythmic delta/theta activity, *LGI1* leucine-rich, glioma inactivated 1, *LP* lumbar puncture, *Ma1* Ma1-protein, *Ma2* Ma2-protein, *MOG* myelin-oligodendrocytes-glycoprotein, *MRI* magnetic resonance imaging, *NMDAR* N-methyl-D-aspartate receptor, *OCBs* oligoclonal bands, *PNS* paraneoplastic neurological syndromes, *Ri* Initials of first patient diagnosed, *RIA* radioimmunoassay, *SOX1* Sry-like high mobility group box 1, *TG* thyroglobulin, *TPO* thyroid peroxidase, *TR[DNER]* Delta/Notch-likeEpidermal growth factor-related Receptor, *TSHR* thyroid-stimulating hormone receptor, *VGKC* voltage-gated potassium channel, *WBC* white blood cell, *WM* white matter, *Yo* initials of first patient diagnosed, *Zic4* Zinc-finger of the cerebellum protein 4.

^a^Anti-DPPX antibodies have been analyzed since approximately September 2018 (in only 150 cases).

^b^Here, the authors only describe earlier published findings^14,24^, no rare and unsystematically recorded new findings.

^c^Anti-Tr- and anti-Zic4-antibodies have been analyzed since approximately August 2015 (in only 453 cases).

### Statistical analyses

Statistical analysis was performed using the Statistical Package for the Social Sciences, version 24 (IBM Corp., Armonk, NY, USA). The results are largely presented in a descriptive manner. Independent sample *t*-tests were used for the comparison of dimensional variables between the subgroups of patients without age difference. ANCOVA analyses with age correction were used to compare all other dimensional variables (e.g., CSF protein concentration between patients with schizophreniform and affective syndromes) between the subgroups with age difference. Categorical variables (e.g., sex) were compared using Chi^2^ tests. A binary logistic regression was performed for age-dependent categorical variables (e.g., number of positive OCBs) between different aged groups. Correlations between CSF basic parameters (WBC count, protein, AQ, and IgG index) with EEG-IRDA/IRTA rates, laboratory results (T3/T4, TSH), clinical findings (number of suicide attempts and number of earlier inpatient stays), and psychometric scores (GAF, CGI, AMDP-scores) were analyzed using Spearman correlation. For correlation analyses, all patients were analyzed together. A p-value of <0.05 was defined as statistically significant for group comparisons and correlation analyses. Due to the exploratory approach of statistical analyses, no correction was performed for multiple testing.

## Results

### Description of the study population

**Fig. 1:**
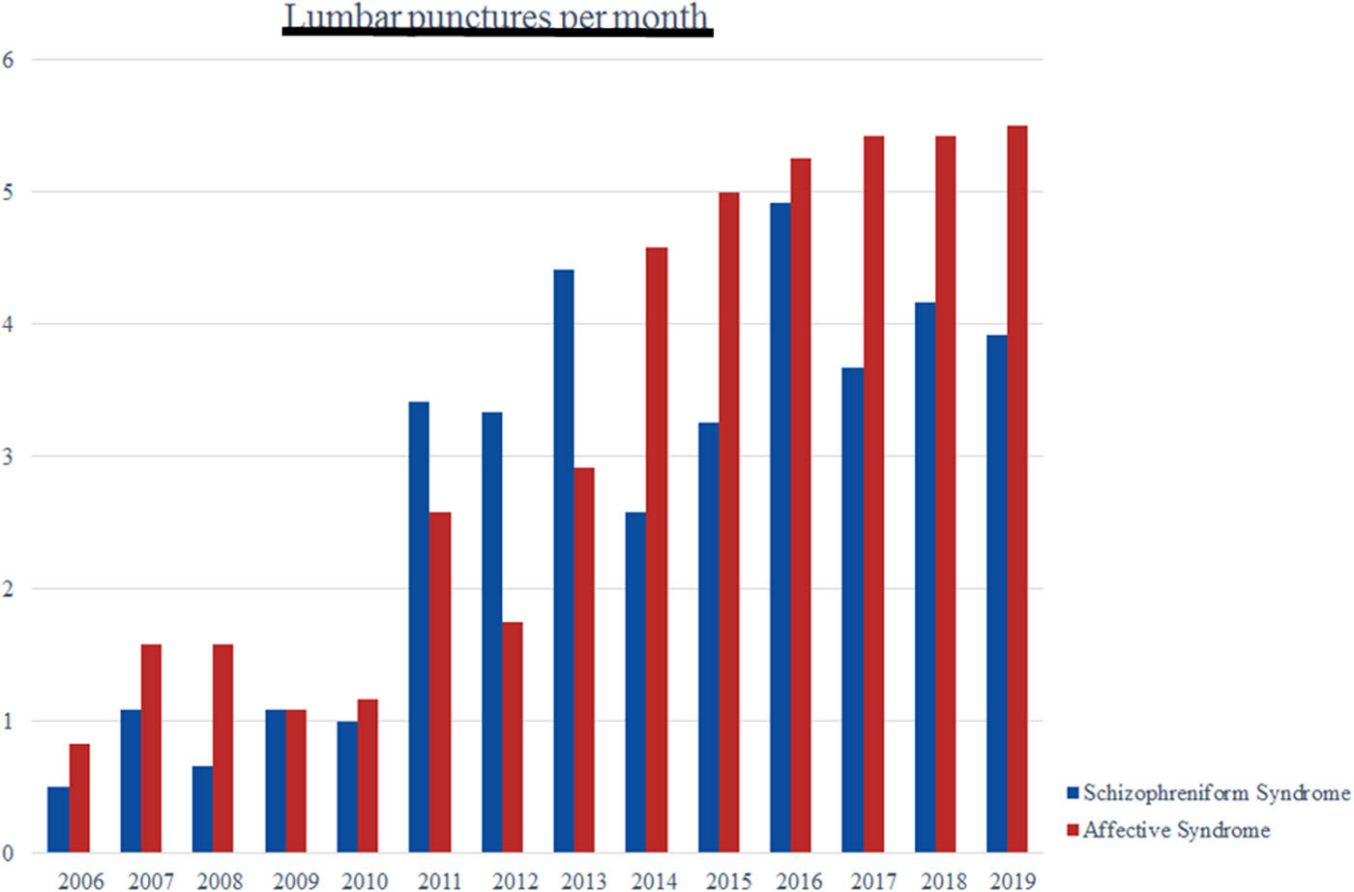
Average number of lumbar punctures per month over the years. If patients have been treated several times as inpatients or have had repeated lumbar punctures, only the first lumbar puncture appears here.

A total of 992 patients were analyzed. Overall, 456 patients presented with schizophreniform syndromes (46%) and 536 with affective syndromes (54%; 455 with unipolar depression and 81 with bipolar disorder). The two subgroups differed significantly in age (*p* < 0.001). The detailed findings are summarized in Tables 2 and 3. The increase in LPs during the study period is summarized in Fig. 1.

**Table 2:** Description of the study sample.

	Total (*N* = 992)	Schizophreniform syndrome (*N* = 456)	Affective syndrome (*N* = 536)	Statistics
*Sociodemographic and clinical findings*				
Sex	445 male (45%):	208 male (46%):	237 male (44%):	Chi^2^ = 0.195
	547 female (55%)	248 female (54%)	299 female (56%)	*p* = 0.659
Age (range) in years	42.75 ± 17.93 (from 18-90)	35.30 ± 14.89 (from 18 to 90)	49.10 ± 17.87 (from 18-90)	*F* = 21.110 ***p*** **<** **0**.**001**
Syndrome^a^	Schizophrenia spectrum (*N* = 456) (46%) Depressive spectrum (*N* = 455) (46%) Bipolar spectrum (*N* = 81) (8%)	Paranoid-hallucinatory: 238 (52%) Hebephrenic: 16 (4%) Catatonic: 10 (2%) Delusional disorders: 25 (5%) Schizoaffective: 122 (27%) - Depressive: 89 (73%) - Manic: 23 (19%) - Mixed: 10 (8%) Acute polymorphic psychotic: 25 (5%) Schizotypal: 3 (1%) Substance-induced psychosis: 7 (2%) Coenesthetic: 3 (0.7%) Undifferentiated/atypical: 3 (0.7%) Prodromal stage: 4 (0.9%)	Mild episode: 2 (0.5%) Moderate episode: 30(7%) Severe episode: 379 (92%) - with psychotic symptoms: 76 (20%) - without psychotic symptoms: 297 (80%) Unknown: 44 (10%) Bipolar, currently depressive: 48 (59%) Bipolar, currently manic: 19 (23%) Bipolar, currently mixed: 14 (17%)	**-**
Clinical course				-
First episode	279 (28%)	188 (41%)	91 (17%)	
Chronic (>2 years)	259 (26%)	122 (27%)	137 (26%)	
Recurrent	445 (45%)	145 (32%)	300 (57%)	
Unknown	9	1	8	
Previous/current comorbid psychiatric disorders				
Neurodevelopmental disorders (ADHD, autism, tic disorder)	89 (9%)	4 (0.9%)	85 (16%)	
Personality disorders	43 (4%)	1 (0.2%)	42 (8%)	
Substance abuse/dependence	114 (11%)	4 (0.9%)	110 (21%)	
Anxiety	33 (3%)	0 (0%)	33 (6%)	
OCD	24 (2%)	1 (0.2%)	23 (4%)	
PTSD	18 (2%)	0 (0%)	18 (3%)	
Cognitive disorders (MCI)	41 (4%)	1 (0.2%)	40 (7%)	
Sleep disturbances	18 (2%)	1 (0.2%)	17 (3%)	
Eating disorders	16 (2%)	1 (0.2%)	15 (3%)	
Somatoform disorder	32 (2%)	0 (0%)	32 (6%)	
Others^b^	9 (0.9%)	1 (0.2%)	8 (1%)	
Previous/current comorbid neurological disorders				
Neurovascular	24 (2%)	5 (1%)	19 (4%)	
Demyelinating	3 (0.3%)	1 (0.2%)^c^	2 (0.4%)^d^	
Extrapyramidal/movement disorders	16 (2%)	3 (1%)	13 (2%)	
Infectious	6 (1%)	1 (0.2%)	5 (1%)	
Tumors	3 (0.3%)	0 (0%)	3 (0.6%)	
Paroxysmal disorders	19 (2%)	8 (2%)	11 (2%)	
Traumatic injuries	23 (2%)	17 (4%)	6 (1%)	
Polyneuropathy	20 (2%)	1 (0.2%)	19 (4%)	
Migraine and other headache	46 (5%)	16 (4%)	30 (6%)	
Restless Legs Syndrome	16 (2%)	1 (0.2%)	15 (3%)	
Hydrocephalus	11 (1%)	3 (0.7%)	8 (1%)	
Others	22 (2%)	5 (1%)	17 (4%)	
*Psychopharmacological treatment*				
Overall psychopharmaco-logical treatment				-
Yes	916 (94%)	429 (96%)	487 (93%)	
No	56 (6%)	20 (4%)	36 (7%)	
Unknown	20	7	13	
Antidepressants				-
Overall	518 (57%)	116 (27%)	402 (83%)	
Tricyclic	59 (6%)	11 (3%)	48 (10%)	
SSRI, SNRI, NDRI, NARI	451 (49%)	105 (24%)	346 (71%)	
MAO inhibitors	8 (0.9%)	0 (0%)	8 (2%)	
Antipsychotics				-
Overall	671 (73%)	412 (96%)	259 (53%)	
“Typical”	142 (16%)	82 (19%)	60 (12%)	
Low-potency	100 (11%)	52 (12%)	48 (10%)	
Medium-potency	0 (0%)	0 (0%)	0 (0%)	
High-potency	42 (5%)	30 (7%)	12 (2%)	
“Atypical”	620 (68%)	393 (92%)	227 (47%)	
Mood stabilizers				-
Lithium	149 (16%)	35 (8%)	114 (23%)	
Anticonvulsants	143 (16%)	75 (17%)	68 (14%)	
Benzodiazepines	146 (16%)	75 (17%)	71 (13%)	-
Number of psycho-pharmacological medi-cation classes per patient				-
Same class/only one drug	378 (41%)	191 (45%)	187 (38%)	
Two drugs	325 (35%)	150 (35%)	175 (36%)	
Three drugs	165 (18%)	70 (16%)	95 (20%)	
Four drugs	45 (5%)	15 (3%)	30 (6%)	
Five drugs	3 (0.3%)	3 (0.7%)	0 (0%)	

*ADHD* attention deficit hyperactivity disorder, *PTSD* post-traumatic stress disorder, *OCD* obsessive-compulsive disorder, *MCI* mild cognitive impairment, *SSRI* selective-serotonin-reuptake-inhibitor, *SNRI* serotonin-noradrenalin-reuptake-inhibitor, *NDRI* norepinephrine-dopamine-reuptake-inhibitor, *NARI* noradrenalin-reuptake-inhibitor, *MAO* monoamine oxidase.

^a^If the lumbar puncture was conducted after clinical improvement, patients were attributed to the initial clinical syndrome.

^b^Other psychiatric comorbidity: dissociative disorders; somatoform disorders include somatization disorders, hypochondriac disorders, persistent pain disorders. ^c^Relapse of multiple sclerosis with pure psychiatric manifestations.

^d^Multiple sclerosis has been initially diagnosed.

Significant *p*-values are marked in bold.

**Table 3:** Psychometric and clinical data of the study sample.

	Total (*N* = 992)	Schizophreniform syndrome (*N* = 456)	Affective syndrome (*N* = 536)	Statistics
*Clinical information*				
Suicide attempts				-
None	282 (64%)	103 (62%)	179 (64%)	
One	108 (24%)	37 (23%)	71 (25%)	
Two	30 (7%)	12 (7%)	18 (6%)	
Three	9 (2%)	4 (2%)	5 (2%)	
Four	4 (0.9%)	3 (2%)	1 (0.4%)	
Five	5 (1%)	3 (2%)	2 (0.7%)	
Six	2 (0.5%)	1 (0.6%)	1 (0.4%)	
Seven	1 (0.2%)	1 (0.6%)	0 (0%)	
>Seven	1 (0.2%)	0 (0%)	1 (0.4%)	
Unclear	548	292	256	
Earlier inpatient stays				-
None	147 (21%)	71 (23%)	76 (19%)	
One	183 (26%)	68 (22%)	115 (28%)	
Two	126 (18%)	57 (19%)	69 (17%)	
Three	89 (12%)	38 (12%)	51 (13%)	
Four	42 (6%)	15 (5%)	27 (7%)	
Five	51 (7%)	17 (6%)	34 (8%)	
>Five	75 (11%)	42 (14%)	33 (8%)	
Unclear	279	148	131	
School education				-
No degree	14 (2%)	9 (3%)	5 (1%)	
Low degree	174 (21%)	70 (20%)	104 (23%)	
Medium degree	235 (29%)	103 (29%)	132 (29%)	
High degree	384 (47%)	169 (47%)	215 (47%)	
Other	8 (1%)	5 (1%)	3 (1%)	
Unknown	177	100	77	
Occupation				-
Employed	266 (32%)	96 (28%)	170 (34%)	
House-wife/-husband	28 (3%)	13 (4%)	15 (3%)	
Unemployed	126 (15%)	61 (18%)	65 (13%)	
Disability pension	101 (12%)	41 (12%)	60 (12%)	
Retirement pension	133 (16%)	13 (4%)	120 (24%)	
In-training/in studies/retraining	149 (18%)	98 (29%)	51 (10%)	
Others	28 (3%)	16 (5%)	12 (2%)	
Unknown	161	118	43	
*Psychometric scores*				
GAF	44.08 ± 69.78	47.92 ± 106.95	41.36 ± 14.29	*F* = 8.050
Unknown	185	121	64	*p* = 0.189
CGI				-
Borderline ill	5 (1%)	5 (1%)	0 (0%)	
Mildly ill	28 (3%)	26 (7%)	2 (0.4%)	
Moderately ill	113 (13%)	83 (22%)	30 (6%)	
Markedly ill	181 (21%)	64 (17%)	117 (22%)	
Severely ill	477 (56%)	169 (46%)	308 (24%)	
Extreme severely ill	50 (6%)	24 (6%)	26 (5%)	
Unknown	138	86	50	

*GAF* global assessment of functioning, *CGI* clinical global impression.

### Cerebrospinal fluid basic findings

WBC counts were increased in 4% of the patients (range from 1 to 101/µL: 87% ≤30/µL, 11% ≤100/µL, 3% >100/µL), IgG indices were increased in 2%, OCBs were detected in 10% (in 4% CSF specific), AQs were elevated in 18%, and protein concentration was elevated in 45% (range from 107 to 2890 mg/L). Therefore, 8% of the patients discerned signs of neuroinflammation (i.e., increased WBC counts/IgG indices and/or CSF specific OCBs), and 18% revealed signs of blood–brain-barrier (BBB) dysfunction with increased AQs. Overall, 50% of the patients displayed some level of CSF alteration (including elevated protein levels). CSF protein levels were more frequently increased in patients with affective disorders (Wald = 5.571, *p* = 0.018; Table 4).

**Table 4:** Cerebrospinal fluid findings.

	Total (*N* = 992)	Schizophreniform syndromes (*N* = 456)	Affective syndromes (*N* = 536)	Statistics
*Cerebrospinal fluid basic parameters*				
WBC counts (Mean ± SD, range)	1.97 ± 4.85 (from 1 to 101/µl)	2.11 ± 6.46	1.84 ± 2.81	*F* = 0.228 *p* = 0.633
Increased WBC counts (ref. <5 /µl)	↑: 38 (4%) ↔: 944 (96%) n.a.: 10	↑: 15 (3%) ↔: 439 (97%) n.a.: 2	↑: 23 (4%) ↔: 505 (96%) n.a.: 8	Wald = 1.771 *p* = 0.183
Protein concentration (Mean ± SD, range)	471.71 ± 238.71 (from 107 to 2890 mg/l)	459.75 ± 230.92	481.86 ± 244.89	*F* = 1.079 *p* = 0.299
Increased protein concentration (ref. < 450 mg/l)	↑: 448 ↑ (45%) ↔: 543 = (55%) n.a.: 1	↑: 201 ↑ (44%) ↔: 254 = (56%) n.a.: 1	↑: 247 ↑ (46%) ↔: 289 = (54%) n.a.: 0	*β* = 0.341 Wald = 5.571 ***p*** **=** **0**.**018**
Albumin quotients (Mean ± SD)	5.81 ± 3.18	5.64 ± 3.18	5.95 ± 3.19	*F* = 2.682 *p* = 0.102
Increased albumin quotients (ref.: <40y.: < 6.5 × 10^-3^; 40–60y.: <8 × 10^−3^; >60y.: <9.3 × 10^−3^)	↑: 174 ↑ (18%) ↔: 815 (82%) n.a.: 3	↑: 85 ↑ (19%) ↔: 371 = (81%) n.a.: 0	↑: 89 ↑ (17%) ↔: 444 = (83%) n.a.: 3	Chi^2^ = 0.640 *p* = 0.424
IgG-Index (Mean ± SD)	0.50 ± 0.10	0.51 ± 0.11	0.50 ± 0.08	*F* = 0.566 *p* = 0.452
Number of patients with increased IgG indices (ref. <0.7)	↑: 19 (2%) ↔: 970 (98%) n.a.: 3	↑: 8 ↑ (2%) ↔: 448 (98%) n.a.: 0	↑: 11 ↑ (2%) ↔: 522 (98%) n.a.: 3	Wald = 0.028 *p* = 0.866
Isolated OCB in CSF	40 (4%) n.a.: 26	19 (4%) n.a.: 7	21 (4%) n.a.: 19	Wald = 0.029 *p* = 0.865
OCBs in CSF and Serum	52 (5%) n.a.: 27	20 (4%) n.a.: 7	32 (6%) n.a.: 20	Wald = 1.915 *p* = 0.166
OCBs overall	93 (10%) n.a.: 26	39 (9%) n.a.: 7	54 (10%) n.a.: 19	Wald = 1.084 *p* = 0.298
*Cerebrospinal fluid overall variables*				
Inflammatory CSF changes^a^	Yes: 78/992 (8%) No: 914 / 992 (92%)	34/456 (7%) 422/456 (93 %)	44/536 (8%) 492/536 (92%)	Wald = 0.198 *p* = 0.656
Overall basic CSF alterations^b^	Yes: 492/992 (50%) No: 500/992 (50%)	222/456 (49%) 234/456 (51%)	270/536 (50%) 266/536 (50%)	*β* = 0.336 Wald **=** 5.510 ***p*** **=** **0**.**019**

*WBC* white blood cell, *ref.* reference; *n.a.* not available; *OCBs* oligoclonal bands, *CSF* cerebrospinal fluid, *SD* standard deviation.

^a^Inflammatory CSF changes: WBC counts increased and/or IgG indices increased and/or CSF specific oligoclonal bands.

^b^Overall basic CSF alterations: Inflammatory CSF changes and/or increased albumin quotients and/or increased protein concentrations. Abbreviations: WBC, white blood cell; ref., reference; n.a., not available; OCBs, oligoclonal bands, CSF, cerebrospinal fluid; SD, standard deviation.

Significant *p*-values are marked in bold.

Patients with pronounced signs of a BBB dysfunction were further analyzed. Thirty percent (*N* = 7/23) of the patients with a greatly increased protein concentration of >1000 mg/L (5% or *N* = 23/448 of the patients with increased protein concentrations and 2% or *N* = 23/991 of all patients) suffered from schizophreniform and 70% (*N* = 16/23) from affective syndromes. Comparing the age of patients with protein concentrations >1000 mg/L (*N* = 23; M = 50.48±16.21 years) and all patients with protein levels <1000 mg/L (*N* = 968; M = 42.59 ± 17.93 years) highlighted significant differences (*F* = 1.012, *p* < 0.037). When comparing the patients with protein concentrations >1000 mg/L (*N* = 23) and all patients with protein levels <1000 mg/L (*N* = 968), no significant differences in the rate of EEG (Wald = 1.497, *p* = 0.221) or MRI alterations (Wald = 0.196, *p* = 0.658) were found. In terms of inflammatory CSF alterations (WBC count, IgG Indices, CSF specific OCBs), significant differences (Wald = 9.187, *p* = 0.002) were detected with higher rates in patients with protein concentrations >1000 mg/L (26%; vs. 7% in patients with protein concentration <1000 mg/L). There were differences in the number of earlier suicide attempts with higher rates in patients with protein concentrations >1000 mg/L (64%) versus patients with protein concentration <1000 mg/L (35%; Wald = 4.307, *p* = 0.038), but no differences in the number of earlier patient instays, and different psychopathological scores (GAF/AMDP scores). Among the patients with elevated age-related AQs (*N* = 174/989), 49% (*N* = 85/174) suffered from schizophreniform and 51% (*N* = 89/174) from affective syndromes. Their average age was 42.88 (±16.43) years. When comparing the patients with elevated AQs and those with normal AQs (*N* = 815/989), no differences in the rate of EEG/MRI and inflammatory CSF abnormalities were found. Patients with elevated age-related AQs had higher rates of earlier suicide attempts (in 47%) compared with patients with normal AQs (34%; Chi^2^ = 4.143, *p* = 0.042).

The group of patients with first-episode schizophreniform syndromes (*N* = 188) showed the following alterations: increased WBC count in 8/188 patients (4%), elevated AQs in 31/188 patients (16%), increased protein concentration in 74/187 patients (40%), increased IgG indices in 4/188 patients (2%), and CSF-specific OCBs in 11/186 patients (6%). OCBs in serum and CSF were detected in 8/186 patients (4%). Patients with first-episode (M = 32.72 years, SD = 15.59, *N* = 188) and recurrent/chronic (M = 37.07 years, SD = 14.14, *N* = 267) schizophreniform syndromes differed significantly in age (*F* = 0.287, *p* = 0.002). No significant differences were noted in mean WBC count (*F* = 0.214, *p* = 0.644), protein concentration (*F* = 0.070, *p* = 0.791), AQ (*F* < 0.001, *p* = 0.990), and IgG indices (*F* = 0.110, *p* = 0.741), or rate of CSF-specific OCBs (Wald = 2.099, *p* = 0.147) between patients with first-episode schizophreniform syndromes (*N* = 188) and patients suffering from recurrent/chronic schizophreniform syndromes (*N* = 267).

Patients with (M = 42.88 years, SD = 18.04, *N* = 916) and without (M = 43.00 years, SD = 17.09, *N* = 56) psychotropic drugs did not differ significantly in age (*F* = 0.758, *p* = 0.960). The BBB dysfunction (increased AQs) showed no statistically significant differences between patients with (*N* = 164/913; 18%) and patients without (*N* = 10/56; 18%) psychotropic drugs (Chi^2^ = 0.000, *p* = 0.984).

Schizophreniform patients with (*N* = 59, 13%) and without (*N* = 397, 87%) neurological comorbidities did not differ significantly in age (*F* = 6.254, *p* = 0.226) and showed no statistically significant differences regarding signs of BBB dysfunction (increased AQs) (Chi^2^ = 1.157, *p* = 0.282) or in terms of inflammatory CSF pathologies (Chi^2^ = 1.909; *p* = 0.167). Depressive/bipolar patients with (M = 57.01 years, SD = 16.70, *N* = 144, 27%) and without (M = 46.19, SD = 17.41, *N* = 392, 73%) neurological comorbidities differed significantly in age (*F* = 1.993, *p* < 0.001) also showed no significant differences in terms of signs of BBB dysfunction (increased AQs; Wald = 1.665, *p* = 0.197) or in the rate of inflammatory CSF changes (Wald = 2.034, *p* = 0.154).

### Autoantibody testing

The detailed Ab findings are summarized in Table 5. Anti-thyroid Abs were detected in 17% of all patients.

**Table 5:** Autoantibody findings.

Autoantibody findings	Overall (*N* = 992)	Schizophreniform syndrome (*N* = 456)	Affective syndrome (*N* = 536)	Statistics
*IgG anti-thyroid antibodies in SERUM against the following antigens*				
TPO (reference: <34 IU/ml)	↑: 72 (17%), ↔: 341 (83%) n.a.: 579	↑: 34 (14%), ↔: 201 (86%) n.a.: 221	↑: 38 (21%), ↔: 140 (79%) n.a.: 358	Chi^2^ = 3.331 *p* = 0.068
TG (reference: <115 IU/ml)	↑: 37 (15%), ↔: 210 (85%) n.a.: 745	↑: 20 (13%), ↔: 129 (87%) n.a.: 307	↑: 17 (17%), ↔: 81 (83%) n.a.: 438	Chi^2^ = 0.715 *p* = 0.398
TSHR (reference: <1.75 IU/l)	↑: 11 (2%), ↔: 469 (98%) n.a.: 512	↑: 5 (2%), ↔: 238 (98%) n.a.: 213	↑: 6 (3%), ↔: 231 (97%) n.a.: 299	Chi^2^ = 0.120 *p* = 0.729
Anti-thyroid antibodies overall	**↑: 91 (17%), ↔: 439 (83%)** n.a.: 462	**↑: 44 (16%), ↔: 230 (84%)** n.a.: 182	**↑: 47 (18%), ↔: 209 (82%)** n.a.: 280	Chi^2^ = 0.493 *p* = 0.483
*Established IgG antineuronal autoantibodies against the following SERUM antigens (Euroimmun® fixed cell assays - screening)*				
NMDAR	++: 1 (0.2%) +++: 2 (0.4%)	++: 1 (0.5%) +++: 2 (0.9%)	0 (0%)	–
LGI1	++: 1 (0.2%) +++: 1 (0.2%)	++: 1 (0.5%)	+++: 1 (0.4%)	–
CASPR2	+: 1 (0.2%)	+: 1 (0.5%)	0 (0%)	–
AMPA-1/2-R, GABA-B-R, DPPX	0 (0%)	0 (0%)	0 (0%)	–
Cell surface antibodies in serum				
Questionably positive	1/475 (0.2%)	1/216 (0.5%)	0/259 (0%)	
Slightly positive	2/475 (0.4%)	2/216 (0.9%)	0/259 (0%)	
Clearly positive	3/475 (0.6%)	2/216 (0.9%)	1/259 (0.4%)	Chi^2^ = 3.513
OVERALL	**6/475 (1%)**	**5/216 (2%)**	**1/259 (0.4%)**	*p* = 0.061
*Established IgG antineuronal antibodies against the following CSF antigens (Euroimmun® fixed cell assays - screening)*				
NMDAR	+: 2 (0.3 %)	+: 2 (0.6 %)	0 (0%)	–
AMPA-1/2-R, GABA-B-R, LGI1, CASPR2, DPPX	0 (0%)	0 (0%)	0 (0%)	–
Cell surface antibodies in CSF				Chi^2^ = 2.134
Questionably positive	**2/741 (0.3%)**	**2/359 (0.6%)**	**0/382 (0%)**	*p* = 0.144
*Established IgG antineuronal antibodies against the following intracellular antigens in SERUM (Ravo® immunoassay - screening)*				
GAD65	+: 2 (0.2%) +++: 1 (0.1%)	+: 1 (0.2%)	+: 1 (0.2%) +++: 1 (0.2%)	–
Amphiphysin	+: 1 (0.1%)	0 (0%)	+: 1 (0.2%)	–
Hu, Ri, Tr(DNER)	0 (0%)	0 (0%)	0 (0%)	–
Yo	+: 7 (0.8%) +++: 2 (0.2%)	+: 5 (1%) +++: 2 (0.5%)	+: 2 (0.5%)**	–
Cv2(CRMP5)	+: 5 (0.6%)	+: 1 (0.2%)	+: 4**/*** (1%)	–
HuD	+: 2 (0.2%) +++: 1 (0.1%)	+: 1 (0.2%)	+: 1** (0.2%) +++: 1* (0.2%)	–
Ma1/Ma2	+ (Ma2): 1 (0.1%) +++ (Ma1): 1 (0.1%)	0 (0%)	+ (Ma2): 1 (0.2%)***** +++ (Ma1): 1 (0.2%)	–
SOX1	+: 17 (2%) +++: 1 (0.1%)	+: 8 (2%)******	+: 9 (2%)**/***/****/***** +++: 1 (0.2%)*	–
Zic4	+: 6 (1%)	+: 1 (0.5%)******	+: 5 (2%)**/***/****/*****	–
Intracellular antibodies in serum				
Questionably positive	**31/826 (4%)**	**16/405 (4%)**	**15/421 (4%)**	Chi^2^ = 0.086 *p* = 0.769
Clearly positive	**5/826 (0.6%)**	**2/405 (0.5%)**	**3/421 (0.7%)**	Chi^2^ = 0.164 *p* = 0.685
*IgG SERUM antibodies associated with demyelinating diseases (Euroimmun® fixed cell assays)*				
AGP4	0 (0%)	0 (0%)	0 (0%)	-
MOG	+++: 1 (1%)	+++: 1 (1%)	0 (0%)	Chi^2^ = 0.528 *p* = 0.468
*Antibody findings in tissue tests (indirect immune-fluorescence on unfixed murine brain tissue: Prof. Prüss, Berlin, Germany)*				
In Serum overall	7 (23%)	5 (31%)	2 (14%)	-
Anti-granule cell pattern	4 (13%)	3 (19%)	1 (7%)	
Anti-vessel pattern	2 (7%)	1 (6%)	1 (7%)	
Anti-myelin pattern	1 (3%)	1 (6%)	0 (0%)	
In CSF overall	8 (27%)	6 (38%)	2 (14%)	
Anti-granule cell pattern	4 (13%)	3 (19%)	1 (7%)	
Anti-vessel pattern	3 (10%)	2 (13%)	1 (7%)	-
Anti-myelin pattern	1 (3%)	1 (6%)	0 (0%)	
Positive tissue tests in serum and/or CSF OVERALL	**9/30 (30%)**	**7/16 (44%)**	**2/14 (14%)**	Chi^2^ = 3.087 *p* = 0.079

+: Questionably positive, ++: Slightly positive, +++: Clearly positive.

*One patient had two +++ antibody findings: HuD and SOX1.

**One patient had multiple + antibody findings: Zic4, Sox1, Yo, CV2, and HuD.

***One patient had multiple + antibody findings: Zic4, Sox1, CV2(CRMP5).

****One patient with two slightly positive antibody findings: anti-SoX1 and Zic4.

*****One patient had multiple + antibody findings: anti-Zic4, Sox1, and Ma2.

******One patient had two + antibody findings: Sox1 and Zic4.

The overall alterations are marked in bold.

Established antineuronal Abs against cell surface antigens were detected in the serum of six patients (out of 475 patients tested overall [1%]) and in the CSF of two patients (out of 741 patients tested overall [0.3%]). The two conspicuous CSF samples were only questionably positive. Positive antineuronal Abs against intracellular antigens in serum were detected in six patients (out of 826 patients tested overall [0.7%]). In two of these patients, Abs were also detected in the CSF (not systematically analyzed). Overall, 31 of 826 patients showed slight Ab reactivity against intracellular antigens (4%). One of 102 tested patients was positive for anti-MOG Abs in serum (1%).

Overall, positive established antineuronal Abs were detected in the serum of 12 patients (1% of 826 tested patients), and in the CSF of four patients (0.5% of 741 tested patients). However, not every single Ab was tested in all patients. A tendency was noted for a more frequent presence of serum Abs against cell surface antigens in patients with schizophreniform syndromes (*p* = 0.061).

Tissue tests were performed in 30 patients who showed *red flags* for AP (mean age: 42.87 ± 17.71 years; sex ratio: 14 males and 16 females; eight patients with first-episode schizophreniform syndromes [27%]; eight patients with recurrent/chronical course of schizophreniform psychosis [27%], two patients with first-episode affective syndromes [7%]; 12 patients with recurrent/chronical course of affective syndromes [40%]). Seven patients (23%) had positive results in their serum, and eight patients were positive in their CSF (27%), mostly with predominant IgG binding to cerebellar and/or hippocampal granule cells (for details see Table 5).

Initially, selected samples (*N* = 39) were also examined in the reference laboratory at Oxford. The results have already been published for five positive cases, with low titer anti-VGKC Abs in four patients, and clearly positive anti-NMDAR Abs in one female patient^14,24^.

### Instrument-based diagnostics

The EEG showed abnormalities in 25% of the patients, most frequently as alterations in the form of IRDAs/IRTAs (in 17%). They were significantly more frequent in patients with schizophreniform psychosis (*p* > 0.01). In the automated IRDA/IRTA detection, tendencies for different IRDA/IRTA rates after HV (*p* = 0.075), and for the IRDA/IRTA difference (*p* = 0.066), with higher rates in patients with schizophreniform syndromes, were found. MRIs revealed overall changes in 72% of patients, with the most frequent being non-specific white matter changes (in 42%, including each individual non-specific lesion), in 9% the MRI findings were compatible with (post-)inflammatory changes. The findings are shown in detail in Table 6.

**Table 6:** Instrument-based diagnostics.

	Total (*N* = 992)	Schizophreniform syndrome (*N* = 456)	Affective syndrome (*N* = 536)	Statistics
EEG	*N* = 954 (96%)	*N* = 449 (98%)	*N* = 505 (94%)	
*Visual assessment*				
Continuous generalized slow activity	34 (4%)	19 (4%)	15 (3%)	–
Continuous regional slow activity	6 (0.6 %)	5 (1%)	1 (0.2%)	–
Intermittent generalized slow activity	162 (17%)	103 (23%)	59 (12%)	–
Intermittent regional slow activity	53 (6%)	25 (6%)	28 (6%)	–
Epileptic pattern	29 (3%)	23 (5%)	6 (1%)	–
EEG overall alterations	242/954 (25%)	142/449 (32%)	100/505 (20%)	*β* = 0.709 Wald **=** 18.485 ***p*** **<** **0.001**
*Automatic IRDA/IRTA quantification (mean values per minute)*				
		*N* = 445 (99%)	*N* = 501 (99%)	
IRDA/IRTA rate before hyperventilation	1.73 ± 2.36	1.88 ± 2.56	1.60 ± 2.16	*F* = 0.323 *p* = 0.570
IRDA/IRTA rate after hyperventilation (*N* = 803, 85%)	2.71 ± 3.98	3.04 ± 4.41 (N = 396)	2.39 ± 3.48 (*N* = 407)	*F* **=** 3.182 *p* = 0.075
Difference in IRDA/IRTA rates before and after hyperventilation (N=803, 85%)	0.86 ± 3.08	1.09 ± 3.32 (N = 396)	0.63 ± 2.81 (*N* = 407)	*F* = 3.398 *p* = 0.066
IRDA/IRTA rate overall	1.90 ± 2.50	2.13 ± 2.80	1.70 ± 2.17	*F* = 1.460 *p* = 0.227
*MRI*	*N* = 896 (90%)	*N* = 418 (92%)	*N* = 478 (89%)	
*Visual assessment*				
*White/Gray matter changes overall* ^a^	461 (51%)	172 (41%)	289 (60%)	Wald = 1.148 *p* = 0.284
Non-specific white matter changes	375 (42%)	145 (35 %)	230 (48%)	Wald = 0.347 *p* = 0.556
Gray matter changes of amygdalae, hippocampi, other limbic structures	12 (1 %)	3 (1%)	9 (2%)	Wald = 0. 871 *p* = 0.351
Lesions/alterations	6 (0.7%)	3 (1%)	3 (0.6%)	
Atrophy	5 (0.6%)	0 (0%)	5 (1%)	
Sclerosis	2 (0.2%)	0 (0%)	2 (0.4%)	
Possible/probable/ definite (post-) inflammatory changes	77 (9 %)	23 (6 %)	54 (11 %)	*β* = 0.742 Wald **=** 7.218 ***p*** **=** **0.007**
*Atrophic changes overall*^b^	108 (12%)	32 (8%)	76 (16%)	Wald = 0.116 *p* = 0.733
Generalized cortical atrophy	34 (4%)	9 (2%)	25 (5%)	Wald = 0.918 *p* = 0.338
Localized atrophy	52 (6%)	16 (4%)	36 (8%)	Wald = 0.033 *p* = 0.855
Ventricle enlargement	37 (4%)	14 (3%)	23 (5%)	Wald = 0.492 *p* = 0.483
*Macroangiopathic vascular alterations (post-ischemic changes)*	33 (4%)	7 (2%)	26 (5%)	Wald = 0.388 *p* = 0.533
*Microhaemorrhage*	17 (2%)	3 (0.7%)	14 (3%)	Wald = 1.109 *p* = 0.292
*Cysts, tumors, anatomical variants and other changes*				
Cysts	119 (15%)	63 (15%)	56 (12%)	Wald = 0.210 *p* = 0.647
Pineal cyst	67 (7%)	38 (9%)	29 (6%)	
Arachnoid cyst	28 (3%)	12 (3%)	16 (3%)	
Fissura choroidea cyst	9 (0.7%)	3 (0.7%)	6 (1%)	
Others^c^	25 (3%)	13 (3%)	12 (3%)	
Tumors	13 (1%)	4 (1%)	9 (2%)	Wald = 0.181 *p* = 0.671
Meningioma	7 (0.8%)	2 (0.5%)	5 (1%)	
Cavernoma	5 (0.6%)	2 (0.5%)	3 (0.6%)	
Acusticus neurinoma	1 (0.1%)	0 (0%)	1 (0.2%)	
Pituitary adenoma	3 (0.3%)	2 (0.5%)	1 (0.2%)	
Anatomical variants and other changes	209 (23%)	107 (26%)	102 (21%)	Wald = 1.750 *p* = 0.186
DVA	37 (4%)	20 (5%)	17 (4%)	
Hippocampal malrotation	3 (0.3%)	2 (0.5%)	1 (0.2%)	
Falx metaplasia	2 (0.2%)	2 (0.5%)	0 (0%)	
Others^d^	8 (0.9%)	5 (1%)	3 (0.6%)	
Ventricle changes				
Asymmetries	57 (6%)	31 (7%)	26 (5%)	
NPH aspect	10 (1%)	1 (0.2%)	9 (2%)	
Malformations^e^	5 (0.6%)	2 (0.5%)	3 (0.6%)	
Perivascular space enlargement	16 (2%)	8 (2%)	8 (2%)	
Virchow-Robin’s space enlargement	39 (4%)	13 (3%)	26 (5%)	
Subarachnoid space enlargement	18 (2%)	9 (2%)	9 (2%)	
Megacisterna magna	6 (0.7%)	5 (1%)	1 (0.2%)	
Gliosis of unclear origin	4 (0.4%)	1 (0.2%)	3 (0.6%)	
Others^f^	23 (3%)	13 (3%)	10 (2%)	
*Overall MRI changes*	641 (72%)	273 (65%)	368 (77%)	Wald = 0.031 *p* = 0.861

Several EEG and MRI changes were noted, if existing.

*EEG* electroencephalography, *IRDA/IRTA* intermittent rhythmic generalized delta/theta activity, *MRI* magnetic resonance imaging.

^a^White/gray matter changes overall: non-specific white matter changes and/or gray matter changes of amygdalae, hippocampi, other limbic structures and/or (post-)inflammatory changes.

^b^Atrophic changes overall: generalized cortical atrophy and/or localized atrophy and/or ventricle enlargement.

^c^Neuroepithelial cyst (*N* = 1), neuroglial cyst (*N* = 1), plexus cysts (*N* = 4), hypophysis cysts (*N* = 6), thalamus cyst (*N* = 1), hygromae (N = 4), unspecified cysts (*N* = 8).

^d^Vascular anomalies (*N* = 4), additional sulci (*N* = 3), heteropia (*N* = 1).

^e^Focal cortical dysplasia (*N* = 2), arteriovenous malformation (*N* = 1), hamartoma (*N* = 1), schizencephaly (*N* = 1).

^f^Thalamus lesion (*N* = 2), hypophysis alterations (*N* = 12), cerebellar hypoplasia (*N* = 1), cerebellar lesion (*N* = 1) others (*N* = 3). Abbreviations: EEG, electroencephalography, IRDA/IRTA, intermittent rhythmic generalized delta/theta activity; MRI, magnetic resonance imaging.

Significant *p*-values are marked in bold.

### Description of antineuronal antibody-positive patients

A total of 24 patients were positive for antineuronal Abs (this includes an anti-NMDAR Ab-positive, older case tested at Oxford; however, weakly positive anti-VGKC Ab titers or weak reactivities in the ravo blot® for Abs against intracellular antigens were not considered as positive). This group included significantly more patients with schizophreniform psychoses (*N* = 18; Chi^2^ = 6.577, *p* = 0.010). Overall, 58% of the Ab-positive patients had CSF alterations (signs of inflammation in 22%; increased AQs in 21%), 54% had MRI signs, and 33% had EEG abnormalities. In addition, 60% of the patients examined with FDG-PET (*N* = 9/1 5) displayed abnormalities. In summary, signs of brain involvement were detected in 92% of the clearly Ab-positive cases (one alteration in 33%, two alterations in 29%, three alterations in 25%, and four alterations in 4%). The findings in the clearly Ab-positive patients are presented in detail in Table 7.

**Table 7:** Patients with positive antibody findings.

	Antineuronal antibody	Age, sex	Syndrome	Stage of disease	CSF	EEG	MRI	FDG-PET	Immuno-modulatory treatment outcome
	*Antineuronal antibodies against cell surface antigens*								
1.	Anti-NMDA-R Ab (+++ in serum, − in CSF)^a^	Mid-20s, f	Atypical psychosis (dissociative states)	Relapse	+ (protein ↑)	=	=	n.p.	Ø
2.	Anti-NMDA-R Ab (+++ in serum^b^, ++ follow-up measurement after anti-inflammatory treatment^b^, in CSF not conducted)	~30, f	Catatonia (initially one seizure)	First episode	++ (protein ↑, AQ ↑, externally initially increased WBC count)	++ (slowing)	++ (atrophic changes)	++ (hypometabolic changes)	Rapid improvement with steroids and plasmapheresis, later azathioprine and mycophenolate mofetil
3.	Anti-NMDA-R Ab (+++ in serum, maximum titer: 1:320 (ref. <1:10) ^c^, − in CSF)	~20, f	Acute polymorphic psychotic disorder	First episode	=	++ (slowing)	++ (WM changes)	++ (hyper- and hypometabolic changes)	Rapid improvement with steroids
4.	Anti-NMDA-R Ab (++ in serum, - in CSF)^a^	Mid 20s, f	Paranoid-hallucinatory syndrome (questionable dyscocnitive seizures initially)	First episode	++ (protein ↑, WBC count ↑)	++ (slowing)	=	=	Rapid improvement with steroids
5.	Anti-NMDA-R Ab (− in serum, + in CSF)	Mid 30s, m	Schizoaffective syndrome (mixed type)	Chronic	++ (protein ↑, AQ ↑)	=	++ (WM changes)	n.p.	No relevant improvement with steroids
6.	Anti-NMDA-R Ab (− in serum, + in CSF)	Mid 50s, f	Schizoaffective syndrome	Relapse	=	= (initially slowing prior to inpatient admission)	++ (WM changes)	n.p.	Ø
7.	Anti-LGI1 Ab (+++ in serum, − in CSF)^d^	~ 40, f	Severe depressive episode without psychotic symptoms	Relapse	++ (protein ↑)	=	+ (pineal cyst)	n.p.	Ø
8.	Anti-LGI1 Ab (repeatedly ++ in serum; titer of 1:80 (reference <1:20); ^e^, − in CSF)	~50, m	Paranoid syndrome (one status epilepticus initially)	Chronic	++ (protein ↑)	++/= (initially epileptic activity prior to inpatient admission)	++ (temporal FLAIR hyperintensity right)	++ (hypometabolic changes)	Slight improvement with steroids
9.	Anti-CASPR 2 Ab (+ in serum, − in CSF)^f^	~40, m	Paranoid syndrome (with severe cognitive deficits)	First episode	=	++ (Accelerations and slowing)	=	n.p.	Ø
*Antineuronal antibodies against intracellular antigens*									
10.	Anti-Yo Ab (reapetedly +++ in serum, − in CSF)	Mid 20s, f	Paranoid-hallucinatory syndrome	First episode	=	=	=	++ (hyper- and hypometabolic changes)	Ø
11.	Anti-Yo Ab (initially +++ in serum, ++ follow-up measurement, +++ in CSF^g^)	~ 20, f	Paranoid-hallucinatory syndrome	First episode	=	=	++ (atrophic changes)	+/++ (hypometabolic changes)	Ø
12.	Anti-GAD65 Ab (++ in serum; also +++ in serum (titer: 101 U/ml reference: <0.9 U/ml) and CSF (1.9 U/ml)^h^	~ 20, f	Severe depressive episode with autism	Relapse	=	=	=	n.p.	Ø
13.	Anti-HuD- and anti-SOX1 Abs (+++ in serum, in CSF not conducted)	Mid 60s, f	Depressive episode with severe mnestic deficits	First episode	++ (OCBs in CSF, local IgG-synthesis)	++ (slowing and sharp waves)	+ (atrophy of the left hippocampus)	n.p.	No further relevant improvement with steroids (parallel successful tumor treatment)
14.	Anti-Ma1 Ab (+++ in serum, ++ follow-up measurement, - in CSF)	~50, f	Bipolar spectrum with severe cognitive deficits	Relapse	=/(++) 1–2 identical OCBs in CSF and serum	=	=	=	Slight improvement with steroids
*Antibodies associated with demyelinating diseases*									
15.	Anti-MOG Ab (reapetedly +++ in serum, additional titer: 1:320, reference: <1:20 ^i^, in CSF not conducted)	~50, m	Paranoid-hallucinatory syndrome (with states of confusion)	Relapse	+ (protein ↑)	++/= (disorganized alpha rhythm)	++ (WM changes)	n.p.	Ø
*Antibodies in tissue tests*									
16.	Anti-granule cell pattern (+++ in serum and CSF)	~20, m	Catatonia	First episode	=	+ (spikes in ICA analysis, slowing)	+ (pineal cyst)	=	Improvement with steroids, plasma- pheresis and later rituximab
17.	Anti-granule cell pattern (+++ in serum and CSF)	~60, m	Depressive syndrome	Relapse	+ (OCBs in CSF, intrathecal IgM-synthesis)	+ (slowing)	++ (WM changes)	=	Rapid improvement with steroids, later stable with methotrexate
18.	Anti-granule cell pattern (+++ in serum and CSF)	~20, f	Schizoaffective syndrome	First episode	=	=	+ (pineal cyst)	+ (hypometabolic changes)	Slight improvement with steroids.
19.	Anti-granule cell pattern (+++ in CSF)	mid 30, f	Paranoid-hallucinatory syndrome	Relapse	+++ (protein ↑, AQ ↑, OCBs in CSF)	+++ (slowing, spike waves)	++ (inflammatory lesions)	=	Slight improvement with plasmapheresis
20.	Anti-vascular structure pattern (+++ in serum and CSF)	~70, m	Severe depressive episode	Relapse	=	=	=	+ (hypometabolic changes)	Ø
21.	Anti-granule cell pattern (+++ in serum, − in CSF)	~40, m	Catatonia	First episode	=	=	++ (WM changes)	++ (hypermetabolic changes)	Improvement with steroids and plasma-pheresis
22.	Anti-vascular structure pattern (+++ in serum and CSF)	mid 30s, m	Paranoid-hallucinatory syndrome with catatonic features	Relapse	+++ (protein ↑, AQ ↑, WBC count ↑, intrathecal IgM-synthesis)	=	++ (WM changes)	=	Improvement with steroids
23.	Anti- vascular structure pattern (- in serum, +++ in CSF)	mid 50s, f	Schizoaffective syndrome	Relapse	++ (protein ↑)	=	++ (WM changes, DVA)	n.p.	Ø
24.	Anti-myelin pattern (+++ in serum and CSF)	mid 20s, m	Paranoid-hallucinatory syndrome	First episode	++ (protein ↑, AQ ↑)	++ (IRDAs, spike waves)	=	=/+ (slightly accen-tuated metabolism)	Ø

*f* female, *m* male, *IRTA* intermittent rhythmic theta activity, *WM* White Matter, *DVA* Developmental Venous Anomaly. Antibody grading: −: negative, +: questionably positive, ++: slightly positive, +++: clearly positive. FDG-PET grading: +: slight, ++: moderate, +++: strong.

^a^In the laboratory in Oxford (using live cell based assay [CBA]) negative.

^b^In the reference laboratory in Oxford (using live CBAs) positive, not tested using biochip-assays initially. Additionally slightly positive for anti-SOX1 antibodies in serum.

^c^In an additional measurement externally serum titer was elevated up to 1:160 using CBAs (reference <1:20).

^d^Confirmatory analysis using CBAs externally negative.

^e^Confirmatory analysis using CBAs positive with titer determination.

^f^In the laboratory in Oxford (using radioimmunoassays) testing for anti-VGKC antibodies was negative.

^g^Serum testing using Ravo blot® was positive and CSF was negative, in the Euroimmun immunblot® anti-Yo reactivity was found in serum and CSF, in addition, a weak anti-Ma2-reactivity was found in the serum.

^h^The concentration was measured by radioimmunoassays.

^i^Confirmatory analysis externally using live CBAs positive with titer determination. External testing was performed unsystematically in Laboratory Krone (Bad Salzuflen; Germany) or Laboratory Stöcker (Lübeck, Germany).

The comparison between clearly Ab-positive (*N* = 24, 3%) and all Ab-negative patients (*N* = 844; 97%) revealed no significant differences in age (*F* = 1.719, *p* = 0.209) or sex (Chi^2^ = 0.079, *p* = 0.779). Overall, 58% of the patients with clearly Ab-positive findings (*N* = 24) showed CSF basic alterations compared to 53% in Ab-negative patients (Chi^2^ = 0.282, *p* = 0.595); comparing inflammatory CSF changes yielded a significant difference (Chi^2^ = 6.024, *p* = 0.014) between patients with positive Ab findings (22%) and those with negative findings (8%). In the rate of EEG abnormalities, Ab-positive patients (*N* = 8/24, 33%) did not differ from Ab-negative patients (*N* = 203/819, 25%) (Chi^2^ = 0.908, *p* = 0.341). MRI diagnostics revealed no significant difference in terms of white/gray matter and atrophic changes (Chi^2^ = 0.119, *p* = 0.730) between clearly Ab-positive findings (*N* = 13/24, 54%) and Ab-negative findings (*N* = 427/844, 51%). Comparison between clinical parameters revealed that formal thought disorders were observed more frequently in Ab-positive cases (*F* = 0.122, p = 0.024). No differences were noted in other AMDP scores or in GAF and CGI scores.

Overall, 54 % (*N* = 13/24) of patients with clearly Ab-positive findings received immunomodulatory treatment. Of these patients, 87% (N = 11/13) improved with treatment. The treatment attempts in detail are summarized in Table 7.

### Correlation analyses

AQ was significantly correlated with the overall IRDA/IRTA rates (*r* = −0.082, *p* = 0.012; *N* = 943), and IRDA rates after HV (*r* = -0.077, *p* = 0.029; *N* = 802). AQ was also correlated with CGI score (*r* = 0.069, *p* = 0.043; *N* = 853), number of earlier suicide attempts (*r* = 0.097, *p* = 0.041, *N* = 443), AMDP score for disorientation (*r* = 0.097, *p* = 0.007; *N* = 773), AMDP score for fears and compulsions (*r* = -0.165, *p* < 0.01; *N* = 774), AMDP score for hallucinations (*r* = 0.097, *p* = 0.007; *N* = 773), and AMDP score for ego boundary disturbances (*r* = -0.089, *p* = 0.014, *N* = 774). CSF protein levels were significantly correlated with overall IRDA rates (*r* = -0.074, *p* = 0.023; *N* = 945) and IRDA rates before HV (*r* = -0.073, *p* = 0.025; *N* = 945). CSF protein concentration was also correlated with the number of earlier suicide attempts (*r* = 0.111, *p* = 0.020; *N* = 444), AMDP-score for disorientation (*r* = 0.071, *p* = 0.048; *N* = 776), AMDP score for fear and compulsion (*r* = -0.151, *p* < 0.01; *N* = 777), and AMDP score for hallucinations (*r* = −0.082, p = 0.023; *N* = 776). The IgG index was significantly correlated with the difference in IRDA/IRTA rate before and after HV (*r* = 0.072, *p* = 0.042, *N* = 802).

## Discussion

This study describes the multimodal diagnostic assessment of a large group of patients with schizophreniform and affective psychoses in a naturalistic inpatient setting in a tertiary care hospital. The main CSF results were signs of BBB dysfunction with increased AQs in 18% and inflammatory CSF alterations in 8% of all patients. Positive antineuronal IgG Abs against established intracellular antigens were detected in serum in 0.6% of the patients. Antineuronal IgG Abs against established cell surface antigens were detected in serum of 1% of the patients and in the CSF of 0.3% (CSF samples were only questionably positive). However, patterns of novel antineuronal Abs using tissue tests were detected in the serum and/or CSF of 30% of patients with schizophreniform or affective syndromes and red flags for AP.

The frequent signs of BBB dysfunction in 18% of all patients are consistent with results from a recent meta-analysis of patients with schizophrenia and affective disorders^31^. The BBB dysfunction significantly correlated with more severe symptoms (as measured by suicide attempts and CGI score) in the present sample. Current knowledge does not clarify whether these changes are primarily involved in the pathophysiology of mental illness or whether they are triggered secondarily by psychotropic drugs^32–34^. However, our data rather suggest that the findings are not caused by medication, since the results did not differ significantly between patients with and without psychotropic drug administration. Irrespective of the cause, a disturbance in BBB function can induce a harmful interaction between the innate brain and adaptive peripheral immunity^35^. This, in turn, allows the transfer of antineuronal Abs (e.g., against NMDAR) from the serum to the CSF, thereby leading to anti-brain effects^36^.

Inflammatory changes that included mild pleocytosis, elevated IgG indices, or CSF specific OCBs were also detected in a relevant, subgroup (8%) of all patients. CSF pleocytosis was usually only subtly pronounced (in 87% from 5 to 30/µL); therefore, higher cell counts can be assumed to lead to a fulminant disorder and these patients are not treated on psychiatric wards. With regard to the 8% of patients who showed schizophreniform syndromes with inflammatory changes, the lower prevalence figure compared to some preliminary studies^14,15,23,24,37^ may reflect the screening approach, which has led to an increasing number of patients undergoing LPs over the last few years (see Fig. 1). These inflammatory changes are compatible with pathogen-related pathologies, but they would also be typical for an AE/AP. Both AEs and APs are associated with slightly increased WBC counts or increased IgG indices/CSF specific OCBs^1,2,6,16^.

Antineuronal Ab-associated AEs have recently been described mostly in association with schizophreniform symptomatology^2^. In fact, in the present study samples as well, the detection of serum IgG Abs tended to be more frequent in patients with schizophreniform syndromes. The finding of only a few patients showing questionable CSF Ab positivity is consistent with another study in which 124 patients with schizophreniform psychosis, examined using the same methodology, displayed only negative CSF samples^15^. However, notably, in most of our cases, the serum Ab-positive patients also showed signs of brain involvement in further investigations; indeed, 92% of the Ab-positive patients showed at least one alteration in CSF, MRI, EEG, or FDG-PET findings. Therefore, our assessment is that all patients with antineuronal IgG serum Abs should undergo a careful diagnostic workup to verify possible brain involvement. This holds even if the patients are CSF Ab negative, since isolated serum Abs can still have therapeutic consequences^38^. According to current international consensus criteria, the detection of antineuronal Abs in the serum, in combination with typical EEG or CSF alterations, is indicative of a “probable AP” ^2^.

The role of tissue tests in selected clinical cases is also worth noting and was clinically relevant in several cases. Tissue tests analyzing serum and CSF were frequently conspicuous in patients with *red flags* for AP. Experiences with some patients have already been published^9,39,40^. Besides granule cell patterns, Abs against vascular structures were found, most likely directed against endothelial cells. The significance and specificity of these findings is not yet clear, as data on the prevalence of these findings in healthy controls is lacking. Similar findings were recently described in association with neuromyelitis opticum (NMO) spectrum diseases^41^. Pathophysiologically, Abs directed against endothelial cells might lead to a BBB dysfunction. In this context, different pathophysiological mechanisms could contribute secondarily to the development of psychiatric syndromes^35^. In addition, Abs against myelin structures were found. These findings are interesting in light of the constantly expanding range of NMO spectrum diseases^42^ and their previously described association with psychotic and affective symptoms^43,44^. In summary, our opinion is that novel Abs against so far unknown antigens could play a decisive role in a subgroup of patients with severe mental disorders.

A major limitation of the present study is its retrospective, open, and uncontrolled design, which meant that many patients did not receive all tests. For example, the measurement of established antineuronal Abs against cell surface antigens was not introduced until 2011 in our department. Initially, these Abs were only tested in the CSF, as CSF testing was considered more sensitive for the detection of anti-NMDAR encephalitis^45^. Our own observations revealed that anti-NMDAR encephalitis can probably also occur in patients with isolated positive serum results^38^, so serum analyses were introduced later, in addition to CSF analysis. The retrospective approach of the study also meant that confirmatory test results for the positive Ab findings from other investigators or with other methods or in other laboratories were not routinely performed. In some cases, the questionable Ab-positive cases could not be confirmed externally (e.g., for patient 1 in Table 7), while other findings (e.g., for patient 9 in Table 7) were only slightly positive. However, this is precisely the situation encountered by clinicians in their everyday lives. In the psychiatric setting, weakly positive Ab findings or Ab titers below the current detection threshold of the standard assays could also be relevant. For example, a possibly long-lasting but milder antibody effect on the brain, occurring via processes such as synaptic reconstruction, could lead to subtler psychiatric phenotypes. For this reason, we have openly described all findings, including questionable results and those from external laboratories/follow-up tests, and the additional findings for these patients are summarized in Table 7. Some patients showed constellations of an AP/AE (e.g., case 2 in Table 7), whereas several other cases had assessments that remained more nebulous (e.g., in case 5 in Table 7). In our department, the use of tissue tests was not fully established until the end of 2018. Nevertheless, even now, this very laborious examination remains reserved for selected cases with high suspicion of AP^9,39,40^. The open design, the broad inclusion criteria (e.g., not excluding patients with different comorbidities), and the fact that a tertiary referral center would obviously attract patients for organic differential diagnosis could have led to a distortion of the results. Similarly, the use of an uncontrolled design precluded estimation of the prevalence of CSF alterations and positive tissue tests in healthy individuals. However, comparative values are available from neurological control groups. For example, an increased AQ in patients with retrobulbar neuritis was found in only 3.8%^46^, whereas the authors detected significantly higher percentages (18%) in similarly aged patients with schizophreniform syndromes. Three subgroups of the patients presented here have already been described in the previous studies^14,23,24^. Multimodal prospective screening studies combining all available methods are desirable in future, especially since the sensitivity of different antineuronal Ab test methods differs significantly^47^. These variations in sensitivity may also explain the low Ab prevalence observed in the present study. Unlike some previous large studies analyzing the serum antineuronal Ab prevalence^11,12^, the present study was focused only on IgG Abs that are clearly associated with an AE^48^.

## Conclusions

CSF findings often revealed a dysfunction of the BBB and, less frequently, signs of neuroinflammation. Established high-level antineuronal Abs in serum were rare, and they occurred even less frequently in the CSF. However, several serum-only Ab-positive patients showed evidence of brain involvement in instrument-based clinical studies. Surprisingly, the use of screening tissue tests frequently detected pathologies in pre-selected patients. Novel antineuronal Abs with so far unknown antigens could, therefore, play a decisive role in psychiatry. Further multimodal, prospective, and controlled studies are necessary.
